# Long-Term Survival of Multiple Versus Single Arterial Coronary Bypass Grafting in Elderly Patients

**DOI:** 10.3390/jcm12072594

**Published:** 2023-03-30

**Authors:** Justin Ren, Colin Royse, Nilesh Srivastav, Oscar Lu, Alistair Royse

**Affiliations:** 1 Department of Surgery, University of Melbourne, Melbourne 3050, Australia; 2 Department of Cardiothoracic Surgery, Royal Melbourne Hospital, Melbourne 3050, Australia; 3 Outcomes Research Consortium, Cleveland Clinic, Cleveland, OH 44195, USA; 4 Oxford Medical School, Medical Sciences Division, University of Oxford, Oxford OX1 2JD, UK

**Keywords:** surgery, coronary artery bypass grafting, age, elderly, multiple arterial grafting, total arterial revascularization, radial artery, internal mammary artery

## Abstract

Multiple arterial grafting (MAG) utilizes more than one arterial graft with any additional grafts being saphenous vein grafts (SVG). It remains an infrequently used coronary surgical revascularization technique, especially in elderly patients. Our study aims to evaluate the age-related association with the relative outcomes of multiple versus single arterial grafting (SAG). The Australian and New Zealand national registry was used to identify adult patients undergoing primary isolated CABG with at least two grafts. Exclusion criteria included reoperations, concomitant or previous cardiac surgery, and the absence of arterial grafting. Propensity score matching was used to match patient groups. The primary outcome was all-cause late mortality and the secondary outcomes were 30-day mortality and 30-day hospital readmission. We selected 69,624 eligible patients with a mean (standard deviation) age of 65.0 (10.2) years old. Matching between MAG and SAG generated 16,882 pairs of patients < 70 years old and 10,921 pairs of patients ≥ 70 years old. At a median [interquartile range] follow-up duration of 5.9 [3.2–9.6] years, MAG was associated with significantly reduced mortality compared to SAG (hazard ratio [HR], 0.73; 95% confidence interval [CI], 0.68–0.78; *p* < 0.001) in the younger subgroup as well as the elderly subgroup (HR, 0.84; 95% CI, 0.79–0.88; *p* < 0.001). In conclusion, MAG offers a survival benefit over SAG, in both younger and elderly patients.

## 1. Introduction

Aging predisposes populations to increased prevalence of coronary artery disease (CAD), contributing to a substantial portion of global health and socioeconomic burdens [1]. CAD is also a major cause of mortality and morbidity in elderly populations [2] that often requires myocardial revascularization via percutaneous coronary intervention (PCI) or coronary artery bypass grafting (CABG). PCI is sometimes perceived as a more appropriate and less invasive procedure in elderly patients [3], but a large body of evidence has consistently highlighted the superior long-term clinical benefits associated with the use of CABG regardless of age [4].

CABG has been the preferred revascularization technique, especially for treating multi-vessel and complex coronary artery disease. Even though international guidelines and prevailing evidence advocate multiple arterial grafts (MAG) [5,6,7,8], contemporary surgical practice disproportionately utilizes the left internal mammary artery (LIMA) and supplementary saphenous vein grafts (SVG) [9]. The superior late clinical and angiographic outcomes of LIMA conduit have been well established [10,11], but only recently have radial artery (RA) outcomes been recognized to be superior [12,13,14,15] in comparison to SVG, which undergoes conduit atherosclerosis and progressive failure with 10-year occlusion rate of approximately 50% [16,17].

Since elderly patients have a reduced life expectancy, surgeons often believe that the potential survival benefit of MAG may have insufficient time to be realized. Perhaps a more common barrier to adoption relates to the perceived technical difficulties of MAG [18]. In conjunction with the higher burden of risk factors present in the elderly, the safety and potential benefits of using multiple arteries [19] are frequently considered unimportant or absent. The present study aims to determine whether multiple versus only one arterial graft is associated with better short-term and long-term clinical outcomes in elderly patients.

## 2. Methods

### 2.1. Data Sources

Data from the Australian and New Zealand Society of Cardiac and Thoracic Surgeons (ANZSCTS) registry were used to identify adult patients that received primary isolated CABG with two or more grafts from June 2001 to January 2020. This national database contains information about patients and their procedures that have been prospectively collected and routinely reviewed by the steering committee via multiple administrative links, including the National Death Index. All follow-up on mortality is complete. Surgeries using a single graft or no arterial conduit, surgeries with concomitant cardiac procedures, and reoperations were excluded. A waiver of individual consent was approved by the Melbourne Health Institutional Review Board (#2011.164).

### 2.2. Outcomes

The primary endpoint was long-term all-cause mortality measured from the date of the index operation. The secondary endpoints were binary short-term outcomes, including 30-day mortality and 30-day readmission to the hospital due to a composite of myocardial infarction, arrhythmia, deep sternal wound infection (DSWI), congestive heart failure (CHF) or recurrent angina with each component analyzed separately. In the definition of short-term outcome DSWI patients must have wound debridement with either positive cultures or antibiotic treatment and CHF patients must have either paroxysmal nocturnal dyspnea, deteriorating dyspnea on exertion due to heart failure or appearance of pulmonary congestion on chest X-ray. Upon readmission, the diagnosis of recurrent angina requires an objective confirmation test via angiography, electrocardiogram, exercise test or echocardiogram.

### 2.3. Statistical Analysis

Continuous variables were expressed as mean (standard deviation [SD]) and categorical variables as count (percentage). For all tests, significance was defined as a two-tailed *p* value less than 0.5. The survival and ggplot2 packages were used in R studio, version 4.0.5 (R Foundation for Statistical Computing, Vienna, Austria).

Comparison of long-term survival after CABG between MAG and single arterial grafting (SAG) was conducted using propensity score matching (PSM) to adjust for imbalanced patient demographics, preoperative comorbidities and surgical techniques (Table 1). Variables included age, sex, body mass index, creatinine level (μmol/L), hypertension, hypercholesterolemia, diabetes mellitus, smoking history, dialysis, arrhythmia, cerebrovascular event, peripheral vascular disease, chronic obstructive lung disease, myocardial infarction, left ventricular ejection fraction, congestive heart failure, New York Heart Association classification of heart failures (class I–IV), left main disease, number of grafts, number of diseased vessels, operative status (elective, urgent, emergency and salvation), on-pump and minimally invasive surgery. Matched comparative analyses of MAG vs. SAG were separately conducted in patients younger than 70 years old and patients older than 70 years old at the time of surgery. The weighted Schoenfeld residuals were used to verify the proportionality assumptions. A greedy one-to-one matching algorithm with a caliper width of 0.2 of the SD of the propensity score without replacement was used. In general, a residual mean difference of 10% would demonstrate a balanced covariate between MAG and SAG, indicating adequate matching. A Cox proportional-hazards regression model, including age, was applied to quantify survival differences between matched cohorts via hazard ratios (HRs) with 95% confidence intervals (CIs). The random clustering effect within individual matched pairs was adjusted by sandwich-type robust variance estimation. Kaplan–Meier survival estimates were used to visualize mortality over time. McNemar’s paired *t*-test was used to evaluate secondary binary outcomes for both age groups.

### 2.4. Sensitivity Analysis

Additional survival analysis of the primary endpoint was performed by replacing propensity score matching with a multivariable-adjusted Cox regression hazard model for risk adjustment to ensure the robustness of conclusions. All previous factors were included in this alternative model.

## 3. Results

**Figure 1:**
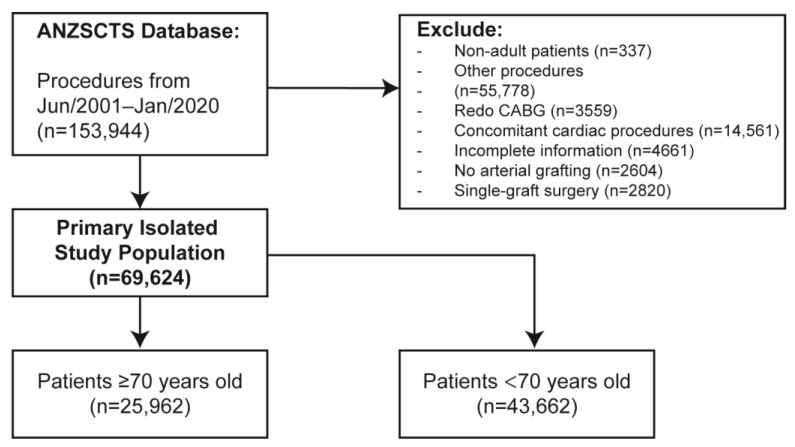
Selection process of eligible patients. ANZSCTS, Australian and New Zealand Society of Cardiac and Thoracic Surgeons; CABG, coronary artery bypass grafting. This flowchart provides information regarding the participant selection process and reasons for exclusions.

In total, we identified 69,624 patients (Figure 1), of which 39,478 (56.7%) were multi-arterial cases and 25,962 (37.3%) were ≥70 years old. There was a mean age (SD) of 65.0 (10.2) years for MAG patients and 67.1 (10.0) years for SAG patients. For the Cox regression model, graphical diagnostics based on weighted Schoenfeld residuals have verified the proportional hazards assumption. Postoperatively, the median follow-up duration was 5.9 years [IQR 3.2–9.6].

### 3.1. Primary Outcome

**Figure 2:**
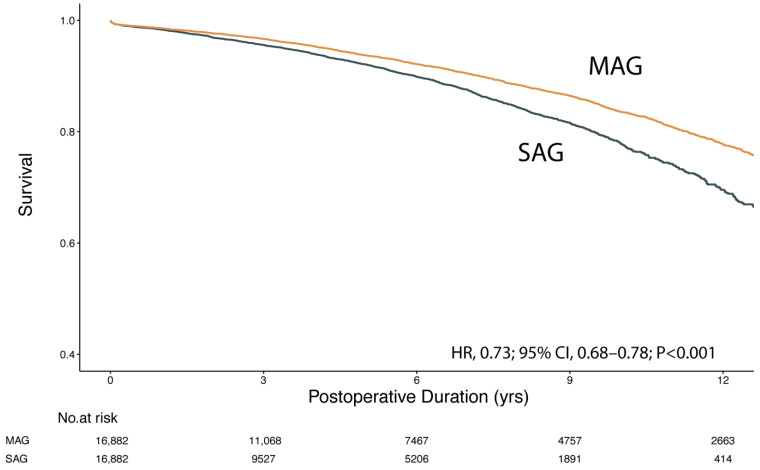
Kaplan–Meier survival curve for MAG vs. SAG in matched cohort <70 years of age. MAG, multiple arterial grafting; SAG, single arterial grafting.

There were 16,882 matched pairs in the cohort younger than 70 years who showed significantly improved long-term survival after multi-arterial revascularization (HR, 0.73; 95% CI, 0.68–0.78; *p* < 0.001). Proportional hazard assumptions of the Cox model were satisfied for all relevant analyses. The Kaplan–Meier curve is presented in Figure 2. There was an incremental divergence of survival curves along the time. The Kaplan–Meier estimated survival rates for MAG were 93.7% at 5 years and 83.6% at 10 years, in comparison to SAG with 92.1% at 5 years and 77.8% at 10 years postoperatively.

**Figure 3:**
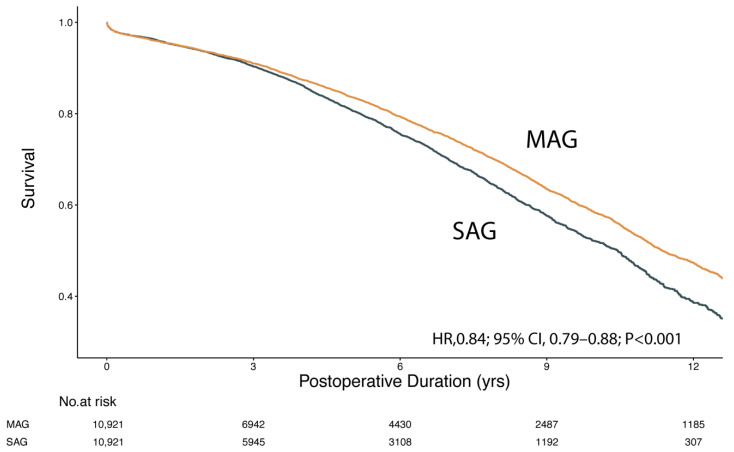
Kaplan–Meier survival curve for MAG vs. SAG in matched cohort ≥70 years of age. MAG, multiple arterial grafting; SAG, single arterial grafting.

A total of 10,921 matched pairs were generated among the cohort aged 70 or older, in which there was again a significant difference in survival between the two groups (HR, 0.84; 95% CI, 0.79–0.88; *p* < 0.001). The Kaplan–Meier curve is shown in Figure 3. The 5-year survival rates for MAG and SAG were 83.7% and 80.8%, respectively, which were further reduced to 58.2% and 52.1% at 10 years postoperatively. Sensitivity analysis using a multivariable-adjusted Cox regression hazard model generated consistent results for both younger (HR, 0.76; 95% CI, 0.70–0.81; *p* < 0.001) and older (HR, 0.87; 95% CI, 0.83–0.92; *p* < 0.001) age groups.

### 3.2. Secondary Outcomes

Within 30 days, MAG procedures in patients older than 70 years old had a significantly higher risk of hospital readmission (*p* = 0.009) and arrhythmia (*p* = 0.007) but a lower risk of myocardial infarction (*p* = 0.009) than SAG. For patients younger than 70 years old, MAG was associated with a significantly reduced incidence of myocardial infarction (*p* < 0.001) and recurrent angina (*p* = 0.005) than SAG. Other 30-day outcomes were similar between the two treatments (Table 2).

## 4. Discussion

This retrospective observational study of a national database reported a previously underexamined impact of age on the relative long-term survival of multiple arterial grafting compared to single arterial grafting. The key finding is that, overall, MAG is associated with improved long-term survival compared to the conventional SAG approach in patients younger than 70 years old, as well as in elderly patients over 70 years old after rigorous statistical adjustment of patients’ perioperative profiles. In younger patients, MAG has reduced 30-day myocardial infarction and recurrent angina. In elderly patients, MAG has reduced 30-day myocardial infarction but has increased hospital readmission and arrhythmia.

The survival curve for MAG appears to diverge from SAG incrementally for patients aged 70 or less, with an absolute difference of 8.5% at 10 years postoperatively that continues to increase along the time. In the elderly cohort, there remains a similar divergent survival difference rising towards a peak of 6.5% at 10 years. The minor reduction in the peak survival difference may be attributable to age but the benefit of MAG is sustained in both cohorts. Unlike the conventional SAG approach, the MAG technique prioritizes the use of arterial conduits in favor of saphenous vein grafts which have well-documented vulnerability to late graft occlusions, thus improving the average graft patency and long-term survival. MAG also seems to have a protective effect against the early development of myocardial infarction and recurrent angina, likely due to the superior patency of arterial conduits. What is troubling and difficult to explain in our study is that we observed an increased incidence of early postoperative arrythmia following multi-arterial procedures. Our current data analysis cannot justify this unexpected finding, but it may relate to any surgical inflammatory factors including endothelial trauma, blood loss or the use of cardiopulmonary bypass. Our observations on survival are in line with those reported by prior investigations. A state-wide propensity-matched study [7] by Rocha and colleagues included a cumulative survival curve of MAG versus SAG up to 8 years postoperatively, documenting an incremental survival advantage of multi-arterial revascularization (HR, 0.80; 95% CI, 0.66–0.97) along the follow-up duration. Another retrospective analysis of the New Jersey State Registry [20] yielded 2882 matched patient pairs and confirmed that MAG, compared to SAG, was associated with a reduced risk of long-term mortality (HR, 0.75; 95% CI, 0.68–0.83) and repeated revascularization (HR, 0.86; 95% CI, 0.76–0.97). Similarly, the MAG cohort from the post hoc analysis of the SYNTAX trial [21] also demonstrated significantly lower incidence of all-cause mortality (adjusted HR, 0.74; 95% CI, 0.55–0.98; *p* = 0.038) compared to the SAG cohort at 12.6-year follow-up. The ART trial [22], of a larger sample size, however, showed no difference in survival between bilateral (BIMA) versus single internal mammary artery (SIMA) revascularization, and could be due to a high cross-over rate and the lack of discrimination between RA and SVG. From the as-treated analysis, the investigators indeed observed a survival difference in favor of MAG.

Aging is associated with increased exposure to risk factors such as hypercholesterolemia, hypertension, obesity and diabetes, leading to increased cellular oxidative stress, inflammatory reactions and modified genetic expressions in the endothelium that lead to endothelial dysfunction, coronary vascular stiffening, progressive atherosclerosis and, thus, CAD [23]. With the proportion of people >60 years old expected to reach 22% by 2050 in developed countries [24], the aging population has become a growing concern and largely increased the incidence of coronary artery disease. The most recent epidemiologic statistics from the America Heart Association Heart Disease and Stroke 2022 Update shows that >20 million adults have CAD, with prevalence exceeding 30% in men and 21.6% in women of >80 years of age, creating enormous health and economic burdens [25]. Improved patient life expectancy has led to an increased cardiovascular disease burden and subsequent upsurge in cardiac interventions [26]. The complicated comorbidities of elderly patients receiving coronary bypass surgery may increase postoperative morbidity and mortality [27,28] compared to younger patients, giving rise to the common perception of PCI as a better treatment option for older patients. However, observational data of patients with multivessel disease from the American College of Cardiology Foundation and The Society of Thoracic Surgeons Collaboration on the Comparative Effectiveness of Revascularization Strategies (ASCERT) study [4] identified reduced mortality for older patients aged 65 or above receiving CABG compared against PCI (risk ratio, 0.79; 95% CI, 0.76–0.82). Another propensity-matched study by Wu et al. [29] documented that the CABG cohort had significantly higher 5-year survival rates than the PCI cohort with drug-eluting stent (HR, 0.71; 95% CI, 0.67–0.77). The subsequent subgroup analysis found a consistent association between the use of CABG with reduced risk of death across all age groups [26].

CABG can be performed safely in elderly patients [30,31], and the attention has now shifted towards finding the optimal surgical configuration. Multi-arterial revascularization has been recommended by the American Heart Association guidelines for patients undergoing isolated CABG [6]. The usual MAG operation utilizes RA and IMA grafting or, alternatively, BIMA. A reduced dependency on SVG, which is associated with lower patency rates and long-term survival, is reported in randomized and meta-analytic series [32,33,34,35,36]. Despite this guideline, of the 281,515 CABG patients recently reported by the Society of Thoracic Surgeons Database, only 14% of patients underwent MAG (8.5% LIMA+RA, 5.6% BIMA). The practice of MAG remains infrequent and seems limited to dedicated institutions, creating a substantial mismatch between the current evidence in the literature and real-world practice. Our finding of improved MAG survival for both young and elderly cohorts supports the routine use of more than one arterial conduit across all age groups.

The presence of survival benefits observed in our elderly MAG population is contrary to conventional understanding. A Canadian study reported a significant association (*p* = 0.002) of age with the survival benefit of MAG relative to SAG. Their risk-adjusted subgroup analyses found improved long-term survival for patients <70 years of age undergoing MAG compared to SAG (HR, 0.76; 95% CI, 0.67–0.85), but a similar survival between two procedures (HR, 0.89; 95% CI, 0.77–1.03) for patients ≥70 years of age [37]. Another matched observational study [38] compared patients receiving RA (MAG) versus SVG only (SAG) and found a lower risk for late death after RA grafting (HR, 0.75; 95% CI, 0.57–0.98; *p* = 0.03). Of primary importance, the protection of RA against mortality gradually declined with increasing age. The authors described 70 years of age as the cut-off threshold for the loss of survival benefits from MAG [38]. In contrast, Navia and colleagues [39] reported that BIMA grafting had superior 10-year survival (HR, 0.66; 95% CI, 0.45–0.97; *p* = 0.036) than procedures with SIMA grafting. As the largest age-stratified MAG study to date, our analysis could overcome sample size limitations which may be responsible for the discordant results in the literature. As one of the major impediments against wider MAG applications, the incidence of deep sternal wound infection in Navia’s study [39] was numerically higher in the BIMA cohort (2.1%) than the SIMA cohort (1.2%), but it appeared that the early adverse effect of sternal infections was not translated into late clinical consequences, even in elderly populations.

Several investigations have proposed that in elderly patients, off-pump procedures may offer better clinical prognosis in comparison to on-pump procedures following CABG [40,41]. A meta-analysis of 14 non-randomized studies [42] showed significantly reduced 30-day mortality (odds ratio [OR], 0.48; 95% CI, 0.28–0.84) in off-pump CABG with even greater advantages in octogenarians (OR, 0.26; 95% CI, 0.12–0.57) compared to on-pump CABG. The incidence of atrial fibrillation was also lower in the off-pump group (OR, 0.77; 95% CI, 0.61–0.97), likely due to less invasive procedures [42]. Another meta-analysis of five randomized controlled trials (n = 6221), however found similar 30-day and mid-term mortality between off-pump and on-pump techniques in the elderly [43]. Additionally, the on-pump cohort exhibited higher early re-intervention (OR, 3.22; 95% CI, 1.28–8.09; *p* = 0.01) and incomplete revascularization rates (34% in off-pump vs. 29% in on-pump; *p* < 0.01) than the off-pump cohort. The contemporary CABG practice involves mostly the conventional on-pump technique (Table 1) because of inconsistent reporting in the literature and unclear benefits of off-pump techniques. More definitive evidence is required to warrant the non-use of cardiopulmonary bypass when performing CABG on elderly patients.

Our study is unique in that it conducts the largest comparative analyses between multiple versus single arterial revascularization in age-specific cohorts, thus examining the subgroup interaction between age and survival difference. The national registry has an official linkage agreement with the National Death Index of Australia that offers near-population coverage, accurate survival data, and variables for comprehensive statistical risk adjustment. We also introduced a sandwich-type robust variable estimation algorithm in our Cox regression hazard model which is able to correct for random clustering effects within the matched pairs themselves and therefore allows for better estimation of treatment effects [44].

### Limitations

This study should be interpreted with recognition of important limitations. Our current dataset did not capture some outcome predictors such as the type and quality of conduits, graft configurations, harvesting techniques, surgeon expertise, degree of preoperative coronary stenosis and the completeness of revascularization, which may all contribute to differential outcomes. Therefore, residual unmeasured confounders may have introduced intrinsic bias that cannot be adequately adjusted for by any statistical methodology. The number of elderly patients was small relative to the overall sample size, which could limit the statistical power to detect significant treatment effects of MAG among patients over 70 years old.

## 5. Conclusions

Multiple arterial grafting conferred superior long-term survival compared to single arterial grafting up to the late period in younger patients and also older patients aged 70 or above. Advanced age should not be a contraindication for using more than one arterial conduit.

## Figures and Tables

**Table 1:** Baseline characteristics of patients undergoing single arterial and multiple arterial coronary artery bypass grafting in Australia before and after propensity score matching.

	Before Matching			After Matching		
**Counts (%)**	**SAG**	**MAG**	**SMD**	**SAG**	**MAG**	**SMD**
**Patients ≥ 70 years old**	12,694	13,268		10,921	10,921	
Age	76.2 ± 4.3	75.9 ± 4.2	0.07	76.0 ± 4.2	76.0 ± 4.3	0.01
Male	9734 (76.7)	10,001 (75.4)	0.03	8269 (75.7)	8231 (75.4)	0.01
Body mass index	28.0 ± 4.9	28.1 ± 4.7	0.02	28.1 ± 4.9	28.1 ± 4.8	0.00
Creatinine (μmol/L)	104.3 ± 71.1	99.8 ± 56.6	0.08	101.0 ± 61.0	100.6 ± 58.6	0.01
Hypertension	10,818 (85.2)	11,058 (83.3)	0.05	9224 (84.5)	9184 (84.1)	0.01
Hypercholesterolemia	10,153 (80.0)	10,578 (79.7)	0.01	8717 (79.8)	8727 (79.9)	0
Diabetes mellitus	4636 (36.5)	4535 (34.2)	0.05	3815 (34.9)	3799 (34.8)	0
*No treatment*	74 (0.6)	66 (0.5)	0.01	63 (0.6)	52 (0.5)	0.01
*Diet control*	645 (5.1)	744 (5.6)	0.02	577 (5.3)	608 (5.6)	0.01
*Oral therapy*	2711 (21.4)	2673 (20.2)	0.03	2256 (20.7)	2217 (20.3)	0.01
*Insulin treatment*	1206 (9.5)	1052 (7.9)	0.06	919 (8.4)	922 (8.4)	0
Smoking History	7374 (58.1)	7629 (57.5)	0.01	6334 (58.0)	6298 (57.7)	0.01
Dialysis	183 (1.4)	62 (0.6)	0.11	82 (0.8)	79 (0.7)	0
Arrhythmia	1769 (13.9)	1643 (12.4)	0.05	1432 (13.1)	1407 (12.9)	0.06
Cerebrovascular event	1829 (14.4)	1845 (13.9)	0.01	1534 (14.1)	1543 (14.1)	0
PVD	1734 (13.7)	1908 (14.4)	0.02	1525 (14.0)	1563 (14.3)	0.01
COPD	1877 (14.8)	1782 (13.4)	0.04	1547 (14.2)	1537 (14.1)	0
Myocardial infarction	6832 (53.8)	6415 (48.4)	0.11	5584 (51.1)	5485 (50.2)	0.02
Left ventricular ejection fraction						
*>60%*	5840 (46.0)	6833 (51.5)	0.11	5349 (49.0)	5462 (50.0)	0.02
*46–60%*	4299 (33.9)	4179 (31.5)	0.05	3591 (32.9)	3486 (31.9)	0.02
*30–45%*	2039 (16.1)	1867 (14.1)	0.06	1600 (14.7)	1620 (14.8)	0.01
*<30%*	516 (4.1)	389 (2.9)	0.07	381 (3.5)	353 (3.2)	0.02
CHF	1833 (14.4)	1720 (13.0)	0.04	1495 (13.7)	1487 (13.6)	0
NYHA ≥ 3	2518 (19.8)	2718 (20.5)	0.02	2192 (20.1)	2255 (20.7)	0.01
Left main disease	4215 (33.2)	3911 (29.5)	0.08	3399 (31.1)	3334 (30.5)	0.01
Number of grafts	3.1 ± 0.8	3.5 ± 1.0	0.36	3.2 ± 0.8	3.3 ± 0.9	0.10
Number of diseased vessels	2.7 ± 0.5	2.7 ± 0.5	0.03	2.7 ± 0.5	2.7 ± 0.5	0.01
*Single-vessel disease*	131 (1.0)	233 (1.8)	0.06	131 (1.2)	161 (1.5)	0.02
*Double-vessel disease*	2839 (22.4)	3010 (22.7)	0.01	2518 (23.1)	2492 (22.8)	0.01
*Triple-vessel disease*	9664 (76.1)	9978 (75.2)	0.02	8233 (75.4)	8224 (75.3)	0
Operative status						
*Elective*	7482 (58.9)	8514 (64.2)	0.11	6784 (62.1)	6853 (62.8)	0.01
*Urgent*	4753 (37.4)	4394 (33.1)	0.09	3806 (34.9)	3744 (34.3)	0.01
*Emergency*	448 (3.5)	354 (2.7)	0.05	326 (3.0)	319 (2.9)	0
*Salvage*	11 (0.1)	6 (0.1)	0.02	5 (0.1)	5 (0.1)	0
On-pump surgery	12,141 (95.6)	12,146 (91.5)	0.15	10,374 (95.0)	10,256 (93.9)	0.04
Minimally invasive	28 (0.2)	188 (1.4)	0.10	28 (0.3)	46 (0.4)	0.01
**Counts (%)**	**SAG**	**MAG**	**SMD**	**SAG**	**MAG**	**SMD**
**Patients < 70 years old**	17,452	26,210		16,882	16,882	
Age	60.4 ± 7.4	59.5 ± 7.5	0.12	60.3 ± 7.4	59.9 ± 7.3	0.06
Male	14,312 (82.0)	22,558 (86.1)	0.12	13,925 (82.5)	14,219 (84.2)	0.05
Body mass index	29.6 ± 5.7	29.4 ± 5.3	0.03	29.6 ± 5.7	29.5 ± 5.4	0.02
Creatinine (μmol/L)	105.8 ± 108.7	92.5 ± 61.8	0.22	95.9 ± 72.5	94.9 ± 71.7	0.02
Hypertension	13,851 (79.4)	19,660 (75.0)	0.10	13,321 (78.9)	13,062 (77.4)	0.04
Hypercholesterolemia	14,138 (81.0)	21,354 (81.5)	0.01	13,665 (80.9)	13,720 (81.3)	0.01
Diabetes mellitus	7321 (42.0)	9259 (35.3)	0.14	6877 (40.7)	6478 (38.4)	0.05
*No treatment*	153 (0.9)	190 (0.7)	0.02	141 (0.8)	131 (0.8)	0.01
*Diet control*	691 (4.0)	1156 (4.4)	0.02	664 (3.9)	706 (4.2)	0.01
*Oral therapy*	3815 (21.9)	5150 (19.7)	0.06	3687 (21.8)	3560 (21.1)	0.02
*Insulin treatment*	2662 (15.3)	2763 (10.5)	0.15	2385 (14.1)	2081(12.3)	0.06
Smoking History	12,109 (69.4)	17,731 (67.7)	0.04	11,705 (69.3)	11,582 (68.6)	0.02
Dialysis	534 (3.1)	183 (0.7)	0.28	192 (1.1)	179 (1.1)	0.01
Arrhythmia	1293 (7.4)	1489 (5.7)	0.07	1189 (7.0)	1045 (6.2)	0.0
Cerebrovascular event	1438 (8.2)	1770 (6.8)	0.06	1346 (8.0)	1284 (7.6)	0.01
PVD	1623 (9.3)	2001 (7.6)	0.06	1495 (8.9)	1416 (8.4)	0.02
COPD	2093(12.0)	2549 (9.7)	0.08	1976 (11.7)	1833 (10.9)	0.00
Myocardial infarction	9860 (56.5)	13,584 (51.8)	0.09	9430 (55.9)	9153 (54.2)	0.03
Left ventricular ejection fraction						
*>60%*	7918 (45.4)	13,467 (51.4)	0.12	7795 (46.2)	8201 (48.9)	0.05
*46–60%*	5773 (33.1)	8493 (32.4)	0.01	5613 (33.3)	5513 (32.7)	0.01
*30–45%*	2858 (16.4)	3438 (13.1)	0.10	2690 (15.9)	2507 (14.9)	0.03
*<30%*	903 (5.2)	812 (3.1)	0.12	784 (4.6)	661 (3.9)	0.04
CHF	2317 (13.3)	2351 (9.0)	0.15	2064 (12.2)	1844 (10.9)	0.05
NYHA ≥ 3	2917 (16.7)	3955 (15.1)	0.05	2709 (16.1)	2679 (15.9)	0.01
Left main disease	4535 (26.0)	6159 (23.5)	0.06	4353 (25.8)	4233 (25.1)	0.02
Number of grafts	3.1 ± 0.9	3.5 ± 1.0	0.34	3.1 ± 0.9	3.3 ± 0.9	0.12
Number of diseased vessels	2.7 ± 0.6	2.7 ± 0.5	0.02	2.7 ± 0.5	2.7 ± 0.5	0.01
*Single-vessel disease*	258 (1.5)	631 (2.41)	0.06	258 (1.5)	335 (2.0)	0.03
*Double-vessel disease*	4131(23.7)	6139 (23.4)	0.01	4042 (23.9)	4044 (24.0)	0.0
*Triple-vessel disease*	12,959 (74.3)	19,356 (73.9)	0.01	12,498 (74.0)	12,427 (73.6)	0.01
Operative status						
*Elective*	10,567 (60.6)	16,557 (63.2)	0.05	10,288 (60.9)	10,500 (62.2)	0.02
*Urgent*	6260 (35.9)	8971 (34.2)	0.03	6023 (35.7)	5870 (34.8)	0.02
*Emergency*	599 (3.4)	671 (2.6)	0.06	558 (3.3)	502 (3.0)	0.02
*Salvage*	26 (0.2)	11 (0.0)	0.05	13 (0.1)	10 (0.1)	0.01
On-pump surgery	16,661 (95.5)	24,279 (92.6)	0.11	16,112 (95.4)	15,966 (94.6)	0.03
Minimally invasive	32 (0.2)	312 (1.2)	0.09	32 (0.2)	49 (0.3)	0.01

SAG, single arterial grafting; MAG, multiple arterial grafting; PVD, peripheral vascular disease; COPD, chronic obstructive lung disease; NYHA, New York Heart Association class; CHF, congestive heart disease; SMD, standardized mean difference. This table provides the count and percentage breakdown of patient demographics, surgical configurations and comorbidities for patients < 70 years old and patients ≥ 70 years old before and after propensity score matching. The mean ± standard deviation is included for continuous variables.

**Table 2:** Short-term outcomes of patients undergoing single arterial and multiple arterial grafting after propensity score matching.

Binary Outcomes	Number of Events (%)		*p*-Value
	SAG	MAG	
**Age group ≥ 70**	10,921	10,921	
30-day mortality	155 (1.4)	151 (1.4)	0.863
30-day readmission (overall)	245 (2.2)	307 (2.8)	**0.009**
*Arrythmia*	96 (0.9)	138 (1.3)	**0.007**
*Congestive heart disease*	62 (0.6)	86 (0.8)	0.766
*Myocardial infarction*	17 (0.2)	4 (0.0)	**0.009**
*Deep sternal infection*	57 (0.5)	65 (0.6)	0.526
*Recurrent angina*	25 (0.2)	22 (0.2)	0.343
**Age group < 70**	16,882	16,882	
30-day mortality	103 (0.6)	98 (0.6)	0.776
30-day readmission (overall)	343 (2.0)	330 (2.0)	0.641
*Arrythmia*	128 (0.8)	126 (0.7)	0.950
*Congestive heart disease*	57 (0.3)	60 (0.4)	0.853
*Myocardial infarction*	24 (0.1)	3 (0.0)	**<0.001**
*Deep sternal infection*	96 (0.6)	115 (0.7)	0.213
*Recurrent angina*	61 (0.4)	33 (0.2)	**0.005**

## Data Availability

The data that support the findings of this study are available from ANZSCTS centralized registry, and therefore the data are not publicly accessible.
